# Salt suppresses IFNγ inducible chemokines through the IFNγ-JAK1-STAT1 signaling pathway in proximal tubular cells

**DOI:** 10.1038/srep46580

**Published:** 2017-04-20

**Authors:** Yohei Arai, Daiei Takahashi, Kenichi Asano, Masato Tanaka, Mayumi Oda, Shigeru B. H. Ko, Minoru S. H. Ko, Shintaro Mandai, Naohiro Nomura, Tatemitsu Rai, Shinichi Uchida, Eisei Sohara

**Affiliations:** 1 Department of Nephrology, Graduate School of Medical and Dental Sciences, Tokyo Medical and Dental University (TMDU), Tokyo, Japan; 2 Laboratory of Immune regulation, School of Life Science, Tokyo University of Pharmacy and Life Sciences, Tokyo, Japan; 3 Department of Systems Medicine, Keio University School of Medicine, Tokyo, Japan

## Abstract

The mechanisms of immunoactivation by salt are now becoming clearer. However, those of immunosuppression remain unknown. Since clinical evidence indicates that salt protects proximal tubules from injury, we investigated mechanisms responsible for salt causing immunosuppression in proximal tubules. We focused on cytokine-related gene expression profiles in kidneys of mice fed a high salt diet using microarray analysis and found that both an interferon gamma (IFNγ) inducible chemokine, chemokine (C-X-C motif) ligand 9 (CXCL9), and receptor, CXCR3, were suppressed. We further revealed that a high salt concentration suppressed IFNγ inducible chemokines in HK2 proximal tubular cells. Finally, we demonstrated that a high salt concentration decreased IFNGR1 expression in the basolateral membrane of HK2 cells, leading to decreased phosphorylation of activation sites of Janus kinase 1 (JAK1) and Signal Transducers and Activator of Transcription 1 (STAT1), activators of chemokines. JAK inhibitor canceled the effect of a high salt concentration on STAT1 and chemokines, indicating that the JAK1-STAT1 signaling pathway is essential for this mechanism. In conclusion, a high salt concentration suppresses IFNγ-JAK1-STAT1 signaling pathways and chemokine expressions in proximal tubules. This finding may explain how salt ameliorates proximal tubular injury and offer a new insight into the linkage between salt and immunity.

The direct linkage of salt with immunity has received attention^1^. It has been demonstrated that high salt intake increased internal salt storage, leading to the exposure of various kinds of cells to a high salt concentration and the cellular immune modulation^2^,^3^,^4^. Therefore, the direct linkage of salt with the immune system has attracted much attention as a new immune-modifying factor, although the detailed mechanisms of immune activation in response to a high salt concentration have only begun to become clearer.

Chronic salt intake has been generally recognized to be a risk factor for progression of kidney disease^5^. High salt intake causes systemic hypertension and aggravates proteinuria and glomerulosclerosis, inducing renal fibrosis through overproduced cytokines^6^,^7^. On the other hand, many clinical studies have indicated that salt loading ameliorates proximal tubular injury, such as contrast-induced nephropathy and cisplatin-induced nephrotoxicity^8^,^9^. In actual fact, saline hydration therapy is the clinical cornerstone for the prevention of contrast-induced nephropathy and cisplatin-induced nephrotoxicity^10^,^11^. These past studies differ in the type of fluid used for hydration, thereby revealing that hydration using a higher salt concentration, regardless of fluid osmolality, has a greater protective effect. Furthermore, an oral administration of salt without fluid administration had also been proven to be beneficial^12^. These proven evidences of salt suppressing tubular injury cannot be explained by the immune activation by salt, indicating the presence of immunosuppression. However, the mechanism of this immunosuppression by salt remains unknown.

In the present study, we found that interferon gamma (IFNγ) inducible chemokines were suppressed by high salt conditions using microarray analysis, and demonstrated that Janus kinase 1 (JAK1)-Signal Transducers and Activator of Transcription 1 (STAT1) signaling pathways play a key role in salt-induced suppression of the chemokines.

## Results

### Suppression of IFNγ inducible chemokines and receptor in mouse kidney with high salt intake

**Figure 1:**
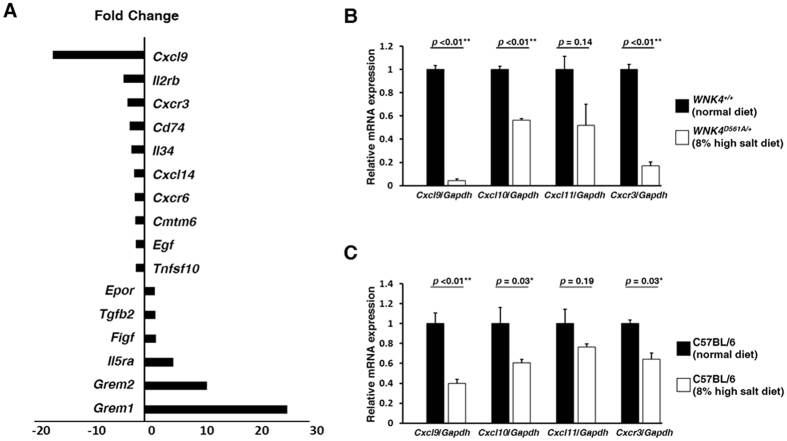
Suppression of IFNγ inducible chemokines and receptor in mouse kidney with excessive salt intake. (**A**) Microarray analyses of the expression of genes related to cytokine and chemokine in the kidneys of the WNK4^D561A/+^ mice fed a high salt diet was compared with those of WNK4^+/+^ littermates fed a normal diet (n = 3). These results demonstrated that both an IFNγ inducible chemokine (Cxcl9, 16.6-fold) and receptor (Cxcr3, 3.0-fold) were dramatically suppressed in the kidneys of WNK4^D561A/+^ mice fed a high salt diet. Values are expressed as fold changes. (**B** to **C**) qRT-PCR analyses of IFNγ inducible chemokines and receptor expression in mouse kidney. (**B**) The comparison between WNK4^D561A/+^ mice fed a high salt diet and WNK4^+/+^ littermates fed a normal diet (n = 3). Not only Cxcl9 and Cxcr3, but also Cxcl10 were suppressed in the kidney of WNK4^D561A/+^ mice fed a high salt diet. (**C**) The comparison between C57BL/6 mice fed a high salt diet with those fed a normal diet (n = 5). The expressions of these Cxcls and Cxcr3 were also suppressed in the kidney of C57BL/6 mice fed a high salt diet. Values are expressed as mean ± standard error of the mean (SEM). *p < 0.05; **p < 0.01.

We use genetically engineered mice with a gain-of-function mutation of with-no-lysine kinase 4 (WNK4), WNK4^D561A/+^ mice, as a mouse model of high salt conditions. WNK4^D561A/+^ mice are impaired urine excretion of salt, caused by an increase in NaCl reabsorption through activation of the thiazide-sensitive Na-Cl cotransporter by WNK4. Using microarray analyses, cytokine-related gene expression profiles in the kidneys of the WNK4^D561A/+^ mice fed a high salt diet were compared with those of WNK4^+/+^ littermates with a normal diet. The results demonstrated that both an IFNγ inducible chemokine (chemokine (C-X-C motif) ligand 9 (CXCL9), 16.6-fold) and a specific receptor (CXCR3, 3.0-fold) were suppressed in the kidney of WNK4^D561A/+^ mice fed a high salt diet (Fig. 1A). Quantitative real time reverse transcription polymerase chain reaction (qRT-PCR) confirmed these findings and further demonstrated that an additional IFNγ inducible chemokine (CXCL10) was also suppressed (Fig. 1B). These CXCLs and CXCR3 were also suppressed in the kidney of C57BL/6 mice fed a high salt diet (Fig. 1C), indicating that the suppressions of CXCLs and CXCR3 were caused by salt loading. These results suggested that salt loading may suppress the expressions of IFNγ inducible chemokines in the kidney and decrease the amount of immune cells possessing an IFNγ related receptor CXCR3.

### Suppression of IFNγ inducible chemokines in proximal tubules caused by salt loading

**Figure 2:**
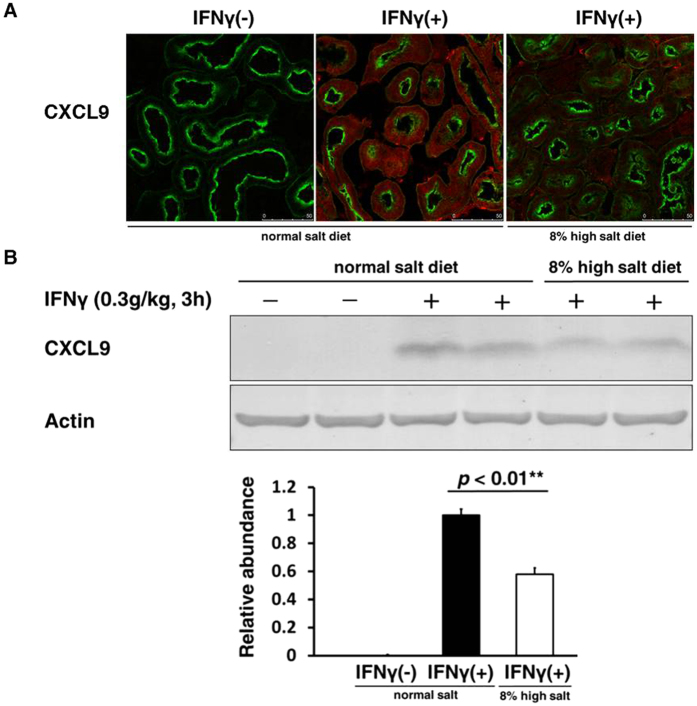
Suppression of IFNγ inducible chemokines in proximal tubules caused by salt loading. To evaluate protein abundances of CXCLs, intraperitoneal injection of recombinant mouse IFNγ (0.3 g/kg) was performed 3 h before organ collection. (**A**) Immunofluorescence staining of CXCL9 induced by IFNγ in kidneys of mice fed a high salt diet compared to those of mice fed a normal diet. The induction of CXCL9 by IFNγ was observed in proximal tubules and was suppressed in kidneys of mice fed a high salt diet. Red; CXCL9, Green; *lotus tetragonolobus* lectin (LTL; proximal tubule marker). Scale bar: 50 μm. (**B**) Upper, Representative immunoblotting performed to evaluate the protein abundance of CXCL9 induced by IFNγ in kidneys of mice fed a high salt diet compared to those of mice fed a normal diet; Lower, Densitometry analysis of the immunoblotting of the CXCL9 (n = 4). Full-length western blot images are presented in Supplementary Figure S1. These results indicated that salt loading suppressed IFNγ inducible chemokines in proximal tubules.

To investigate in which nephron segment the suppression of these CXCLs occurred in kidneys of mice fed a high salt diet, we performed immunofluorescence analysis. The induction of CXCL9 by IFNγ was observed in proximal tubules (Fig. 2A). Immunohistochemistry and immunoblot confirmed the suppression of protein levels of CXCL9 in kidneys of mice fed a high salt diet, indicating that salt loading suppresses IFNγ inducible chemokines in proximal tubules (Fig. 2A,B). These results may coincide with the facts that these CXCLs released from proximal tubules have been proven to exacerbate certain kinds of kidney diseases *in vivo*.

### Suppression of IFNγ inducible chemokines in HK2 cells exposed to a high salt concentration

Renal tubules are often exposed to a high salt concentration by high salt intake based on the countercurrent multiplier system in the kidney. In fact, urinary sodium concentration and excretion of our mice fed a high salt diet were higher than those of mice fed a normal diet, suggesting that renal tubular cells were exposed to high salt condition (Supplementary Tables S1 and 2). Therefore, we investigated whether a high salt concentration suppressed the expression of CXCLs in proximal tubules using HK2 cells. Because naive HK2 cells expressed CXCLs on a low level under measurement sensitivity, we performed the experiments detailed below to investigate the effects of a high salt concentration on the expression of CXCLs under the stimulation of recombinant human IFNγ.

**Figure 3:**
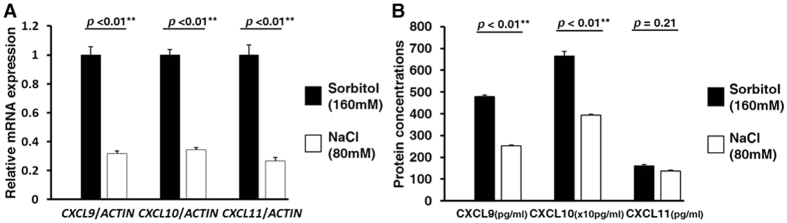
The effect of a high salt concentration on the induction of IFNγ inducible chemokines in HK2 cells. (**A**) qRT-PCR analyses evaluating mRNA expressions of each CXCLs in HK2 cells (n = 4). The cells were treated by IFNγ (10 ng/mL) for 36 h after a 2 h exposure of culture medium supplemented with either NaCl (80 mM) or Sorbitol (160 mM, as an osmotic control). A high salt concentration suppressed mRNA levels of all CXCLs induced by IFNγ, compared to the control. (**B**) ELISA analyses evaluating protein concentrations of each CXCLs released to culture supernatant from HK2 cells (n = 6). The cells were treated by IFNγ (10 ng/mL) for 72 h after a 2 h exposure of culture medium supplemented with either NaCl (80 mM) or sorbitol (160 mM, as an osmotic control). A high salt concentration also suppressed protein concentrations of CXCL9 and CXCL10 induced by IFNγ, compared to the control. Values are expressed as mean ± standard error of the mean (SEM). *p < 0.05; **p < 0.01.

In qRT-PCR analyses, a high salt concentration significantly suppressed messenger RNA (mRNA) levels of CXCL9, CXCL10, and CXCL11 induced by IFNγ, compared to the medium whose osmotic pressure was adjusted to the same level by sorbitol (Fig. 3A). Moreover, enzyme-linked immunosorbent assay (ELISA) analyses of these CXCLs demonstrated that a high salt concentration also suppressed the protein concentrations of CXCL9 and CXCL10 released to culture supernatant from HK2 cells (Fig. 3B). Considering that these CXCLs secreted by epithelial cells have generally been proven to be able to attract immune cells possessing CXCR3, these results suggested that a high salt concentration could block CXCR3-mediated migration of immune cells in proximal tubules, leading to the reduction of CXCR3-mediated proximal tubular injury.

### Suppression of the JAK-STAT signaling pathway in HK2 cells exposed to a high salt concentration

**Figure 4:**
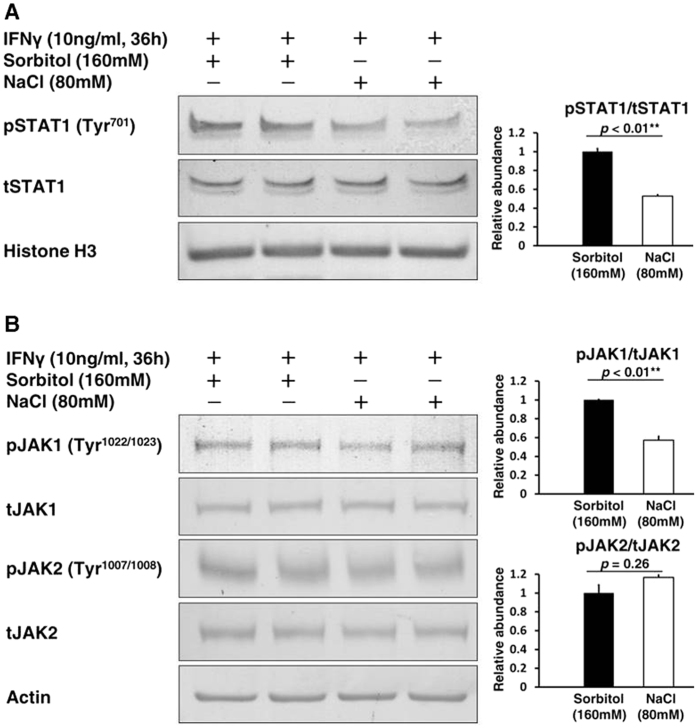
Suppression of the JAK-STAT signaling pathway in HK2 cells exposed to a high salt concentration. The cells were treated by IFNγ (10 ng/mL) for 36 h after a 2 h exposure of culture medium supplemented with either NaCl (80 mM) or Sorbitol (160 mM, as an osmotic control). (**A**) Left, Representative immunoblotting performed to evaluate the phosphorylation of STAT1 in the nucleus; Right, Densitometry analysis of the immunoblotting of the phosphorylation of STAT1 in the nucleus (n = 5). Full-length western blot images are presented in Supplementary Figure S2. A high salt concentration inhibited phosphorylation of STAT1 at tyrosine 701 in the nucleus. (**B**) Left, Representative immunoblotting performed to evaluate the phosphorylation of JAK1 and JAK2; Right-upper, Densitometry analysis of the immunoblotting of the phosphorylation of JAK1 (n = 4); Right-lower, Densitometry analysis of the immunoblotting of the phosphorylation of JAK2 (n = 4). Full-length western blot images are presented in Supplementary Figure S3. A high salt concentration also inhibited phosphorylation of JAK1 at tyrosine 1022/1023, but not phosphorylation of JAK2 at tyrosine 1007/1008.

To understand the molecular mechanisms involved in the suppression of these CXCLs in proximal tubular cells by a high salt concentration, we focused on the JAK-STAT signaling pathway, major up-stream activators of CXCLs, and direct down-stream mediators of the IFNγ signaling pathway. In immunoblottings, a high salt concentration inhibited phosphorylation of an activation site of STAT1 at tyrosine 701 in the nucleus, and thus decreased the transcriptional activity of STAT1 (Fig. 4A). Furthermore, phosphorylation of an activation site of JAK1 at tyrosine 1022/1023 was also inhibited, whereas phosphorylation of an activation site of JAK2 at tyrosine 1007/1008 was not inhibited (Fig. 4B). These results indicated that a high salt concentration suppresses CXCLs through the JAK1-STAT1 phosphorylation cascade in proximal tubular cells.

### A trafficking defect of IFNGR1 to the basolateral membrane in HK2 cells exposed to a high salt concentration

**Figure 5:**
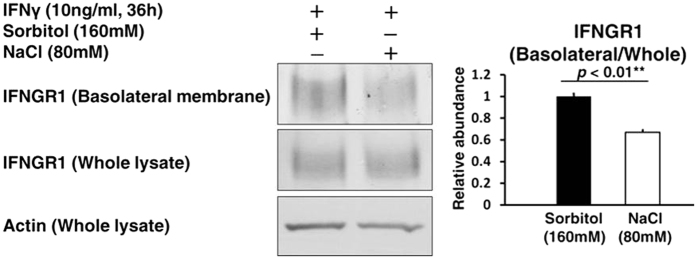
A trafficking defect of IFNGR1 to the basolateral membrane in HK2 cells exposed to a high salt concentration. The cells were treated by IFNγ (10 ng/mL) for 36 h after a 2 h exposure of culture medium supplemented with either NaCl (80 mM) or Sorbitol (160 mM, as an osmotic control). Biotinylation assay was performed to evaluate the protein abundance of IFNGR1 in the basolateral membrane of HK2 cells. Left, Representative immunoblotting performed to evaluate the protein abundance of IFNGR1; Right, Densitometry analysis of the immunoblotting of the protein abundance of IFNGR1 in the basolateral membrane (n = 3). Full-length western blot images are presented in Supplementary Figure S4. Although the protein abundance of IFNGR1 in whole cell lysate was not markedly altered by a high salt concentration, the protein abundance of IFNGR1 in the basolateral membrane significantly decreased.

To explore mechanisms responsible for this signal reduction of the JAK1-STAT1 phosphorylation cascade, we focused on IFNGR1 involved in tyrosine phosphorylation of JAK1. Although IFNGR1 expression in whole cell lysate was not altered by a high salt concentration, the biotinylation assay demonstrated that a high salt concentration significantly decreased IFNGR1 expression in the basolateral membrane of HK2 cells (Fig. 5). These results indicated that the suppression of CXCLs by a high salt concentration results from a trafficking defect of IFNGR1.

### Suppression of JAK1 phosphorylation in mouse kidney with high salt intake

**Figure 6:**
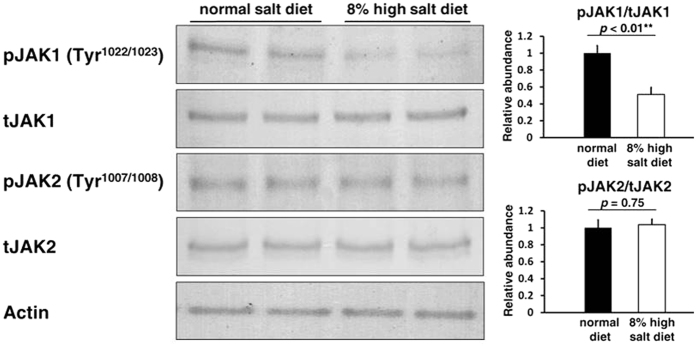
Suppression of JAK1 phosphorylation in mouse kidney with excessive salt intake. The comparison between C57BL/6 mice fed a high salt diet for 7 days and mice fed a normal diet (n = 5). Left, Representative immunoblotting performed to evaluate the phosphorylation of JAK1 and JAK2 in kidney; Right-upper, Densitometry analysis of the immunoblotting of the phosphorylation of JAK1 (n = 4); Right-lower, Densitometry analysis of the immunoblotting of the phosphorylation of JAK2 (n = 4). Full-length western blot images are presented in Supplementary Figure S5. The phosphorylation of JAK1 at tyrosine 1022/1023 was inhibited in the kidney of C57BL/6 mice fed a high salt diet, whereas phosphorylation of JAK2 at tyrosine 1007/1008 was not inhibited.

We confirmed the suppression of the JAK-STAT signaling pathway in mouse kidney with high salt intake. In immunoblottings, the phosphorylation of JAK1 at tyrosine 1022/1023 was inhibited in the kidneys of C57BL/6 mice fed a high salt diet compared to those of mice fed a normal diet. In contrast, phosphorylation of JAK2 at tyrosine 1007/1008 was not inhibited (Fig. 6). These results demonstrated that salt loading certainly suppresses CXCLs through the JAK1-STAT1 phosphorylation cascade *in vivo*.

### The essential involvement of the JAK1-STAT1 signaling pathway in the inductions of IFNγ inducible chemokines

To confirm the essential involvement of the IFNγ inducible JAK1-STAT1 signaling pathway in the regulation of CXCLs by a high salt concentration, we investigated the effect of ruxolitinib, a JAK1 and JAK2 specific inhibitor.

**Figure 7:**
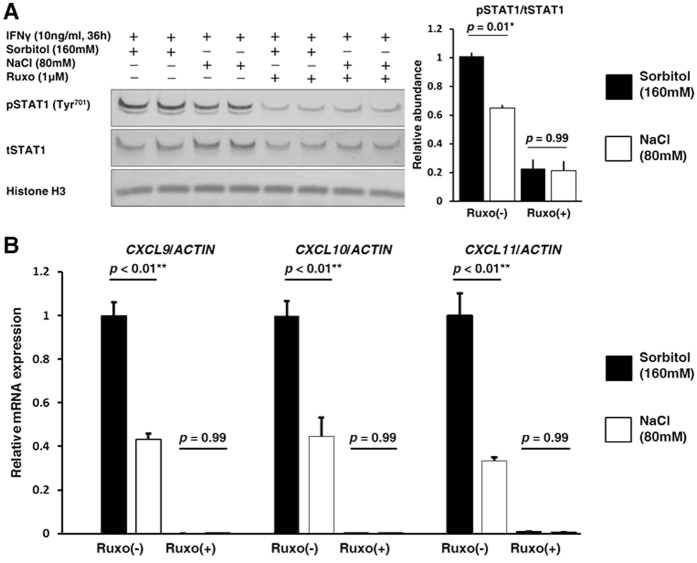
The essential involvement of the JAK1-STAT1 signaling pathway in the inductions of the IFNγ inducible chemokines. The cells were treated by a JAK1 and JAK2 specific inhibitor, ruxolitinib (Ruxo) (1 μM), 2 h before the 36 h of IFNγ (10 ng/mL) treatment. (**A**) Left, Representative immunoblotting performed to evaluate the phosphorylation of STAT1 in the nucleus; Right, Densitometry analysis of the immunoblotting of the phosphorylation of STAT1 in the nucleus (n = 4). Full-length western blot images are presented in Supplementary Figure S6. Ruxo inhibited phosphorylation of STAT1 at tyrosine 701 in the nucleus. (**B**) qRT-PCR analyses were performed to evaluate CXCLs expression (n = 4). The expressions of CXCLs were markedly suppressed and no difference between the high salt concentration condition and control condition was evident.

Ruxolitinib inhibited phosphorylation of STAT1 at tyrosine 701 in the nucleus, eliminating the effect of a high salt concentration on STAT1 (Fig. 7A). In addition, the expressions of CXCLs were suppressed and no difference in expression between the high salt concentration and the control condition was evident (Fig. 7B). These results indicated that the JAK1-STAT1 signaling pathway mainly mediates the suppressive effect of a high salt concentration on the expression of CXCLs.

## Discussion

**Figure 8:**
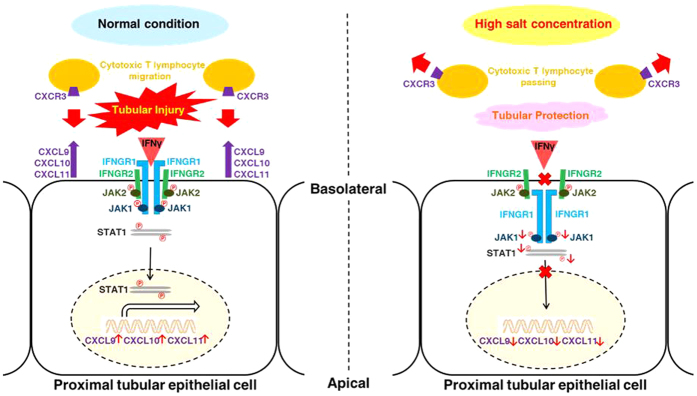
Schematic representation of the mechanism of inhibition of IFNγ-JAK1-STAT1 signaling by the exposure to a high salt concentration in proximal tubular cells. The exposure to a high salt condition decreased the protein abundance of IFNGR1 in the basolateral membrane of proximal tubular cells. This IFNGR1 internalization suppressed phosphorylation of an activation site of JAK1, thereby inhibiting the transcriptional activity of STAT1 through decreasing phosphorylation of its activation sites. Finally, the excretion of IFNγ inducible chemokines from proximal tubular cells was suppressed. Accordingly, the migration of cytotoxic T cells possessing a specific chemokine receptor CXCR3 was presumably decreased.

In the present study, we found that IFNγ inducible chemokines were suppressed in the kidney with high salt intake. We further revealed that a high salt concentration suppressed the expressions of these chemokines in proximal tubules using HK2 cells, indicating the direct effect of salt on the suppression of chemokines. Finally, we demonstrated that a high salt concentration decreased IFNGR1 expression in the basolateral membrane of proximal tubular cells, leading to a decreased phosphorylation of activation sites of JAK1 and STAT1, the up-stream activators of the chemokines. These data indicated that IFNγ-JAK1-STAT1 signaling pathways play a key role in salt-induced suppression of the chemokines (Fig. 8). Our findings could be valuable for further understanding of the effects of salt on the immune system.

We demonstrate that a high salt concentration has a suppressive effect on these IFNγ related immune responses in proximal tubular cells. In fact, renal tubules are often exposed to a high salt concentration by high salt intake. The countercurrent multiplier system in the kidney generates a concentration gradient of salt in the renal medulla^13^. High salt intake increases this salt concentration gradient as much as 800 mEq/L at the medulla, at the location of the proximal tubule S3 segment^14^,^15^. Considering that the tubule S3 segment is the common site of proximal tubular injury, and that tubular injury of the S3 segment is mainly improved by salt loading, a reasonable hypothesis is that the high salt concentration directly affects the proximal tubule at the S3 segment.

This IFNγ related immune response has been demonstrated to be associated with various kidney diseases. IFNγ is a critical cytokine for innate and adaptive immunity, and is mainly secreted by Th1-type CD4^+^ T cells and cytotoxic effector CD8^+^ T cells^16^. Cellular responses to IFNγ are activated through its interaction with a heterodimeric receptor consisting of IFNGR1 and IFNGR2 subunits^17^. Activated IFNGR1 and IFNGR2 phosphorylate JAK1 and JAK2, respectively^18^. Both the activation of JAK1 and JAK2 cooperatively leads to STAT1 phosphorylation, and phosphorylated STAT1 induces various gene transcription processes involved in immune response and cell proliferation. CXCL9 and CXCL10 are generally regarded as IFNγ inducible chemokines^19^,^20^. These CXCLs are secreted from various cells, including proximal tubular cells by stimulation of IFNγ^21^,^22^ and act as chemotactic attractants of immune cells possessing a specific receptor CXCR3^23^. CXCR3 is rapidly induced on naive T cells by stimulation of these CXCLs, and preferentially remains highly possessed on Th1-type CD4^+^ T cells and effector CD8^+^ T cells^24^. Many previous studies demonstrated a role of CXCR3 in the trafficking of Th1 and cytotoxic effector T cells to peripheral sites of Th1-type inflammation^25^,^26^. These CXCLs and CXCR3 are also highly induced on inflamed kidneys and are regarded as a therapeutic target^27^,^28^,^29^.

We consider that the pre-conditioning of salt loading becomes a possible protection mechanism against acute proximal tubular injury through Th1-type inflammation involving these chemokines. In fact, these chemokines have been recognized to be rapidly induced by acute proximal tubular injury within a few hours, and adversely affect the pathological condition during the early acute phase^30^. For example, in a mouse model of acute proximal tubular injury caused by renal microvascular injury, CXCL10 was shown to be rapidly induced in the proximal tubules, and that the expression pattern of CXCL10 overlapped with the pattern of T cell influx^31^. Treatment with a neutralizing CXCL10 antibody reduces the number of infiltrating T cells and improves renal microvascular injury. Moreover, tubulointerstitial nephritis caused by acute rejection of renal allograft induces CXCL9 and CXCL10, and the expressions of these chemokines reflect the severity of proximal tubular injury^32^,^33^. The neutralization of these chemokines using antibody therefore prolongs allograft survival^34^. Furthermore, renal ischemic-reperfusion injury causes acute proximal tubular injury through activated immune cells possessing CXCR3, and CXCR3^−/−^ mice show substantial resistance to kidney injury^35^. In addition, cisplatin-induced nephrotoxicity, one of the most widespread acute proximal tubular injuries, is improved by salt loading^12^,^36^,^37^,^38^, mainly damaging the proximal tubule S3 segment with highly expressed CXCL10^39^,^40^. The exposure to a high salt concentration therefore inhibited cisplatin-induced cytopathy in proximal tubular cells *in vitro*^41^. Considering the above facts that inhibitions of these CXCLs and CXCR3 have protective effect against proximal tubule injury *in vivo* and cultured cells *in vitro*, it is natural to suppose that salt loading ameliorates proximal tubule injury through the inhibition of IFNγ-JAK1-STAT1 signaling pathways and chemokine expression in proximal tubular cells.

In addition, inhibitors of the JAK-STAT signaling pathway may be useful for most proximal tubular injury improved by salt loading. In fact, AG490, a widely prevalent JAK2, but not JAK1, specific inhibitor, has been proven to have renal protective effects on several kinds of proximal tubular injury. In mouse models of renal ischemic-reperfusion injury and cyclosporin A-induced nephrotoxicity, AG490 was shown to decrease proximal tubular injury and improve acute renal failure^42^,^43^. Considering that STAT1 is a common down-stream molecule of JAK1 and JAK2, the inhibition of JAK1 or STAT1 may also suggest a certain degree of renal protective effects and offer an additional treatment approach.

Our results show that IFNGR1 expression in the basolateral membrane is decreased when proximal tubular cells are exposed to a high salt concentration. Many previous studies have reported that several types of receptor internalizations often occurred by cellular stresses, including a high salt concentration^44^,^45^. IFNGR1, but not IFNGR2, has generally been recognized to show this receptor internalization by various stimulations^46^. Although the mechanisms responsible for the IFNGR1 internalization in proximal tubular cells through the exposure to a high salt concentration remains incompletely understood, a receptor internalization specific to IFNGR1 and the subsequent signal reduction of the JAK1-STAT1 phosphorylation cascade are certainly observed by certain kinds of infections^47^.

Although a high salt concentration suppresses the IFNγ related immune response, it must be noted that a high salt concentration also activates a certain kind of immune systems in renal cells. For example, our results of microarray analyses indicated that the expression of Transforming growth factor beta (TGFβ), a major pro-fibrotic cytokine, was increased in a mouse model of high salt conditions. In fact, chronic high salt intake has generally been recognized to cause glomerulosclerosis and tubulointerstitial fibrosis^5^. Therefore, further research is required to determine the situation where salt stimulation has beneficial effects on disease states. Nevertheless, our present research may offer a new insight into the therapeutic possibility of salt.

In conclusion, we demonstrated that salt loading suppresses IFNγ-JAK1-STAT1 signaling pathways and chemokine expression in proximal tubular cells. This finding may explain how salt loading ameliorates proximal tubular injury and offer a new insight into the direct linkage between salt and immunity.

## Methods

### Animals

The generation of the WNK4^D561A/+^ mice and their genotyping strategies were described previously^48^. Studies were performed on each strain using littermates. C57BL/6 mice were purchased from CLEA Japan. The mice were fed a normal diet [0. 4% NaCl (w/w)] or a high salt diet [8.0% NaCl (w/w)] (Oriental Yeast, Japan), and plain drinking water for seven days. In some experiments, to trigger the production of IFNγ inducible chemokines, intraperitoneal injection of recombinant mouse IFNγ (0.3 g/kg, Peprotech) was performed 3 h before organ collection. This experiment was approved by the Animal Care and Use Committee of the Tokyo Medical and Dental University and was performed in accordance with the guidelines for animal experiments of the Ministry of Education, Culture, Sports, Science and Technology, Japan.

### Cell culture

A cultured line of human proximal tubular epithelial cells, HK2 (ATCC; CRL-2190), was cultured in Dulbecco’s Modified Eagle’s Medium/Nutrient Mixture F-12 Ham (DMEM/F-12, 1:1 mixture) supplemented with 10% (v/v) fetal bovine serum (FBS), 100 U per mL penicillin, and 0.1 mg/mL streptomycin. Cells were grown at 37 °C in a humidified incubator with 5% CO_2_. The cells were treated by the addition of recombinant human IFNγ (10 ng/mL, R&D Systems) to trigger IFNγ inducible chemokine ligands. To investigate the effects of a high salt concentration on the expression of these chemokines, culture medium supplemented with either NaCl (80 mM) or Sorbitol (160 mM, as an osmotic control) was used 2 h before the addition of recombinant human IFNγ. In some experiments, ruxolitinib (1 μM, Cayman Chemical), a JAK1 and JAK2 specific inhibitor, was added 2 h before the addition of recombinant human IFNγ.

### Microarray analysis

Total RNA from mouse kidneys was extracted using TRIzol Reagent (Invitrogen) and purified with RNase-free DNase Sets and RNeasy Kits (Qiagen) according to the manufacturer’s protocol. The microarray experiments were performed using SurePrint G3 Mouse Gene Expression 8 × 60 K microarrays (Agilent Technologies). Microarray data files can be obtained from the NIH Gene Expression Omnibus with accession number GSE87600. The data were analyzed by the National Institute on Aging (NIA) microarray analysis tool (http://lgsun.grc.nia.nih.gov/ANOVA/)^49^. Bioinformatics analysis for the gene profile of cytokines and cytokine receptors was performed using the DAVID Functional Annotation Bioinformatics Microarray Analysis (https://david-d.ncifcrf.gov/)^50^,^51^.

### qRT–PCR

Total RNA extracted from mouse kidneys and HK2 cells was reverse-transcribed using ReverTra Ace (TOYOBO, Japan). qRT-PCR analysis was performed in a Thermal Cycler Dice Real Time System (Takara Bio). Primers and templates were mixed with SYBR Premix Ex Taq II (Takara Bio). The amounts of mRNA were normalized to glyceraldehyde 3-phosphate dehydrogenase (GAPDH) or β-ACTIN, and were calculated using the comparative CT method. The primer set for mouse GAPDH was purchased from Takara Bio, and other primer sequences used are summarized in Supplementary Table S3.

### Immunofluorescence

Immunofluorescence was performed as previously described^52^. Mouse kidneys were fixed by perfusion through the left ventricle with 0.2 M periodate lysine and 2% paraformaldehyde in PBS. Tissue samples were soaked for several hours in 20% sucrose in PBS, embedded in Tissue-Tek OCT Compound (Sakura Finetechnical), and snap-frozen in liquid nitrogen. Goat anti-CXCL9 antibody was purchased from R&D Systems. Fluorescent *lotus tetragonolobus* lectin (LTL) was purchased from Vector Laboratories. Alexa fluor (Molecular Probes; Invitrogen) was used for secondary antibodies. Immunofluorescent images were obtained using the Leica TCS SP8 laser-scanning confocal microscope system.

### Immunoblotting

Immunoblotting was performed as previously described^53^. For immunoblotting, we used whole lysates of entire kidney samples without the nuclear fraction (600 g) and crude lysates of HK2 cell samples (15,000 g). Goat anti-CXCL9 antibody was purchased from R&D Systems. Rabbit anti-STAT1 antibody, rabbit anti-phosphorylated STAT1 (Tyr^701^) antibody, rabbit anti-JAK1 antibody, rabbit anti-phosphorylated JAK1 (Tyr^1022/1023^) antibody, rabbit anti-JAK2 antibody, rabbit anti-phosphorylated JAK2 (Tyr^1007/1008^) antibody, and rabbit anti-Histone H3 antibody were purchased from Cell Signaling. Rabbit anti-IFNGR1 antibody was purchased from Santa Cruz Biotechnology. Rabbit anti-β-ACTIN antibody was purchased from Sigma-Aldrich. Alkaline phosphatase-conjugated anti-IgG antibody (Promega) was used as the secondary antibody, and Western Blue (Promega) was used to detect the signals. The band intensities of the western blots were quantified using Image J software (NIH).

### ELISA

The culture supernatant of HK2 cells was obtained 72 h after the addition of recombinant human IFNγ. Each protein concentration of IFNγ inducible chemokine ligand in this supernatant was determined using the appropriate Quantikine ELISA Kit (R&D Systems).

### Biotinylation assay

HK2 cells were seeded on semipermeable filters (Transwell, 0.4 μm pore size; Corning Costar, no. 3412), and cultured for four days with daily changing of the medium. Then, HK2 cells were used for the biotinylation assay 36 h after treatment with recombinant human IFNγ (10 ng/mL, R&D Systems) to the basolateral side of the filters in a 5% CO_2_ incubator at 37 °C. The amount of IFNGR1 in the basolateral membrane was quantitated by basolateral surface biotinylation as previously described^54^.

### Statistics

Statistical significance was evaluated using an un-paired t-test. For multiplex comparisons, the one-way analysis of variance (ANOVA) test with Tukey’s test was used. *P* < 0.05 was considered statistically significant. Data are presented as mean ± standard error of the mean (SEM).

## Additional Information

**How to cite this article**: Arai, Y. *et al*. Salt suppresses IFNγ inducible chemokines through the IFNγ-JAK1-STAT1 signaling pathway in proximal tubular cells. *Sci. Rep.* **7**, 46580; doi: 10.1038/srep46580 (2017).

**Publisher's note:** Springer Nature remains neutral with regard to jurisdictional claims in published maps and institutional affiliations.

## Supplementary Material

Supplementary Information
