# Supplementary material for: Salt suppresses IFNγ inducible chemokines through the IFNγ-JAK1-STAT1 signaling pathway in proximal tubular cells
Source: Sci Rep. 2017 Apr 20;7:46580. doi: 10.1038/srep46580 (PMC5397865; doi:10.1038/srep46580)

Salt suppresses IFNγ inducible chemokines through the IFNγ-JAK1-STAT1 signaling pathway in proximal tubular cells

Yohei Arai[1], Daiei Takahashi[1], Kenichi Asano[2], Masato Tanaka[2], Mayumi Oda[3], Shigeru B. H. Ko[3], Minoru S. H. Ko[3], Shintaro Mandai[1], Naohiro Nomura[1], Tatemitsu Rai[1], Shinichi Uchida[1], and Eisei Sohara[1*]

[1]Department of Nephrology, Graduate School of Medical and Dental Sciences, Tokyo Medical and Dental University (TMDU), Tokyo, Japan.

[2]Laboratory of Immune regulation, School of Life Science, Tokyo University of Pharmacy and Life Sciences, Tokyo, Japan.

[3]Department of Systems Medicine, Keio University School of Medicine, Tokyo, Japan.

*Corresponding author: Eisei Sohara

Department of Nephrology, Graduate School of Medical and Dental Sciences, Tokyo Medical and Dental University (TMDU)

1-5-45 Yushima, Bunkyo, Tokyo 113-8519, Japan

Tel: +81-3-5803-5214; Fax: +81-3-5803-5215; E-mail: esohara.kid@tmd.ac.jp

Blood and urine measurements.

Blood was obtained from the maxillary vein under light ether anesthesia. Blood electrolyte levels were determined with an i-STAT® Portable Clinical Analyzer (Fuso, Japan). The plasma and urine osmolality were measured with a Fiske One-ten Osmometer. Urine electrolyte levels were analyzed by DRI-CHEM (Fujifilm, Japan).

Supplementary Table S1. The comparison of blood and urine analyses between C57BL/6 mice fed a high salt diet for 7days (n = 6) and C57BL/6 mice fed a normal diet (n = 5).

	C57BL/6 normal salt diet (n = 5)	C57BL/6 8% high salt diet (n = 6)	*p*-value
Blood Na concentration (mEq/L)	149.8 ± 0.5	153.6 ± 0.8	<0.01
Urine Na concentration (mEq/L)	274.0 ± 16.3	376.6 ± 21.0	<0.01
Daily urinary Na excretion (mEq/day)	0.43 ± 0.07	5.28 ± 0.12	<0.01
Daily urine output (ml/day)	1.6 ± 0.3	14.2 ± 0.9	<0.01
Plasma osmolality (mOsm/L)	265.2 ± 7.2	263.3 ± 5.0	0.75
Urine osmolality (mOsm/L)	2088.0 ± 214.2	913.5 ± 28.2	<0.01

Supplementary Table S2. The comparison of blood and urine analyses between $WNK4^{D561A/+}$ mice fed a high salt diet for 7days (n = 4) and $WNK4^{+/+}$ littermates fed a normal diet (n = 5).

	$WNK4^{+/+}$ normal salt diet (n = 5)	$WNK4^{D561A/+}$ 8% high salt diet (n = 4)	*p*-value
Blood Na concentration (mEq/L)	149.2 ± 0.86	153.5 ± 1.19	0.01
Urine Na concentration (mEq/L)	260.0 ± 7.0	375.0 ± 37.7	0.01
Daily urinary Na excretion (mEq/day)	0.64 ± 0.05	5.06 ± 0.13	<0.01
Daily urine output (ml/day)	2.4 ± 0.1	13.9± 1.5	<0.01
Plasma osmolality (mOsm/L)	265.7 ± 1.8	267.5 ± 1.8	0.29
Urine osmolality (mOsm/L)	1950.0 ± 67.9	939.0 ± 75.1	<0.01

Supplementary Table S3. The primer sequences used qRT-PCR

**Genes**		Sequences(5-3)
Mouse Cxcl9	Forward	CAC GAT CCA CTA CAA ATC CC
	Reverse	TTC ACA TTT GCC GAG TCC
Mouse Cxcl10	Forward	TTC TTT AAG GGC TGG TCG GAG
	Reverse	GTC GCA CCT CCA CAT AGC
Mouse Cxcl11	Forward	GTT GAA GTG ATT GTT ACT ATG AAG
	Reverse	TGG CAC AGA GTT CTT ATT GG
Mouse Cxcr3	Forward	GCC TGA ACT TTG ACA GAA
	Reverse	GGA AGA GTT AAC ACC AGC
Human CXCL9	Forward	GGA GTG CAA GGA ACC CCA GTA
	Reverse	GGT GGA TAG TCC CTT GGT TGG T
Human CXCL10	Forward	TCC ACG TGT TGA GAT CAT TGC T
	Reverse	TGA TGG CCT TCG ATT CTG GAT
Human CXCL11	Forward	GCT ATA GCC TTG GCT GTG ATA TTG TG
	Reverse	CTG CCA CTT TCA CTG CTT TTA CC
Human β-ACTIN	Forward	TGG CAT TGC CGA CAG GAT GC
	Reverse	TCC ACA CGG AGT ACT TGC GC

Supplementary Figure S1.
Full-length western blot images of the cropped blots shown in Figure 2.
The hatched red lines delineate the correct sized bands for each protein.

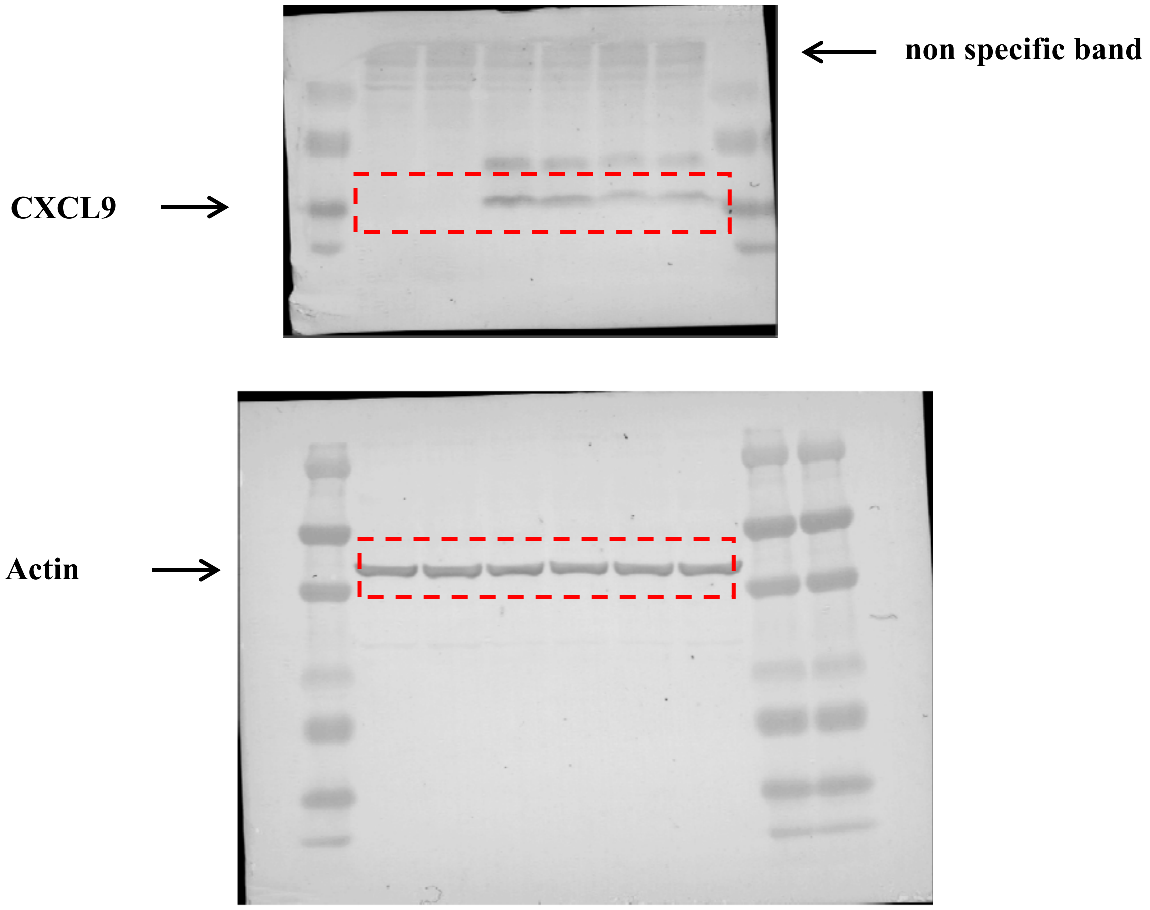

Supplementary Figure S2.
Full-length western blot images of the cropped blots shown in Figure 4A.
The hatched red lines delineate the correct sized bands for each protein.

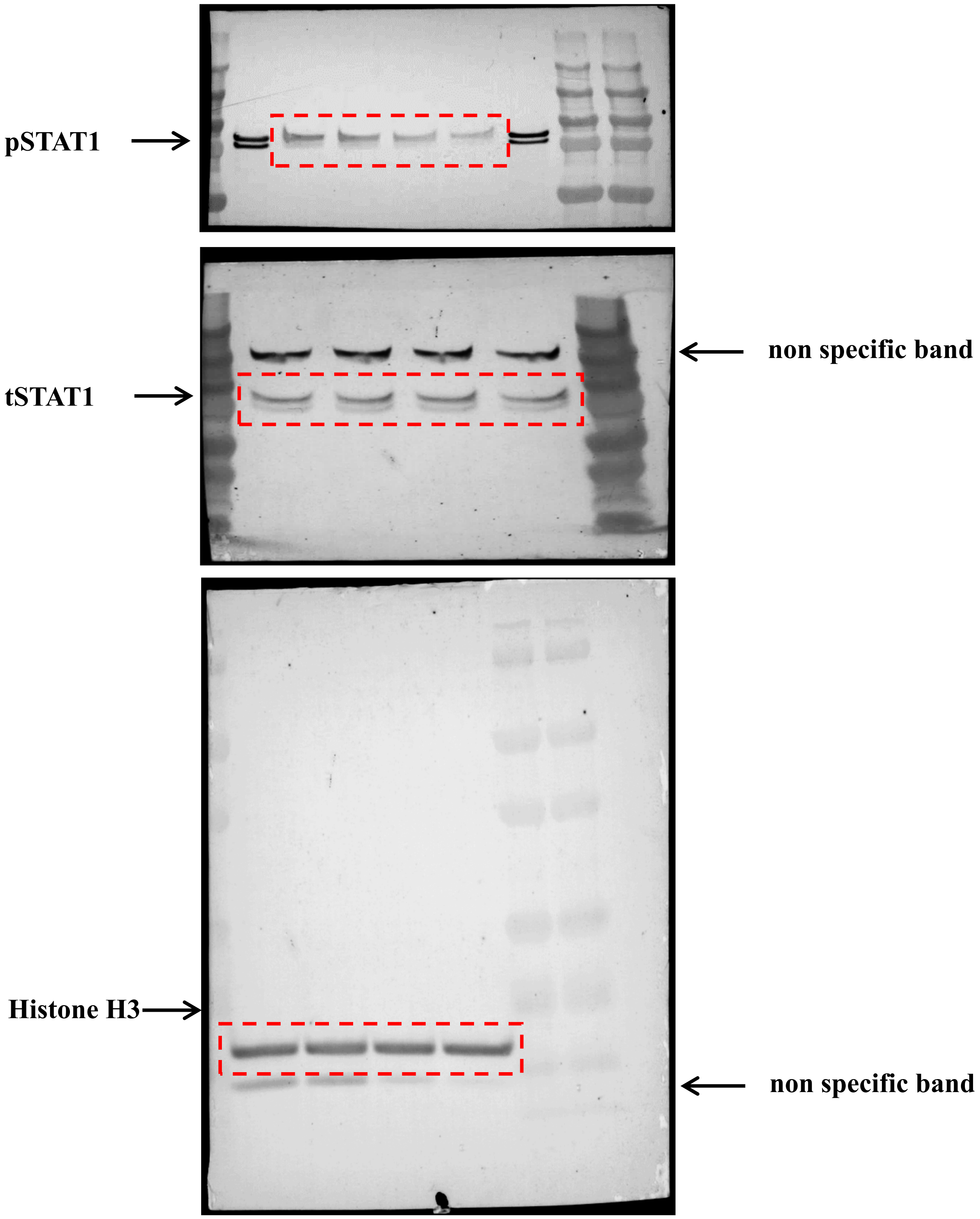

Supplementary Figure S3.
Full-length western blot images of the cropped blots shown in Figure 4B.
The hatched red lines delineate the correct sized bands for each protein.

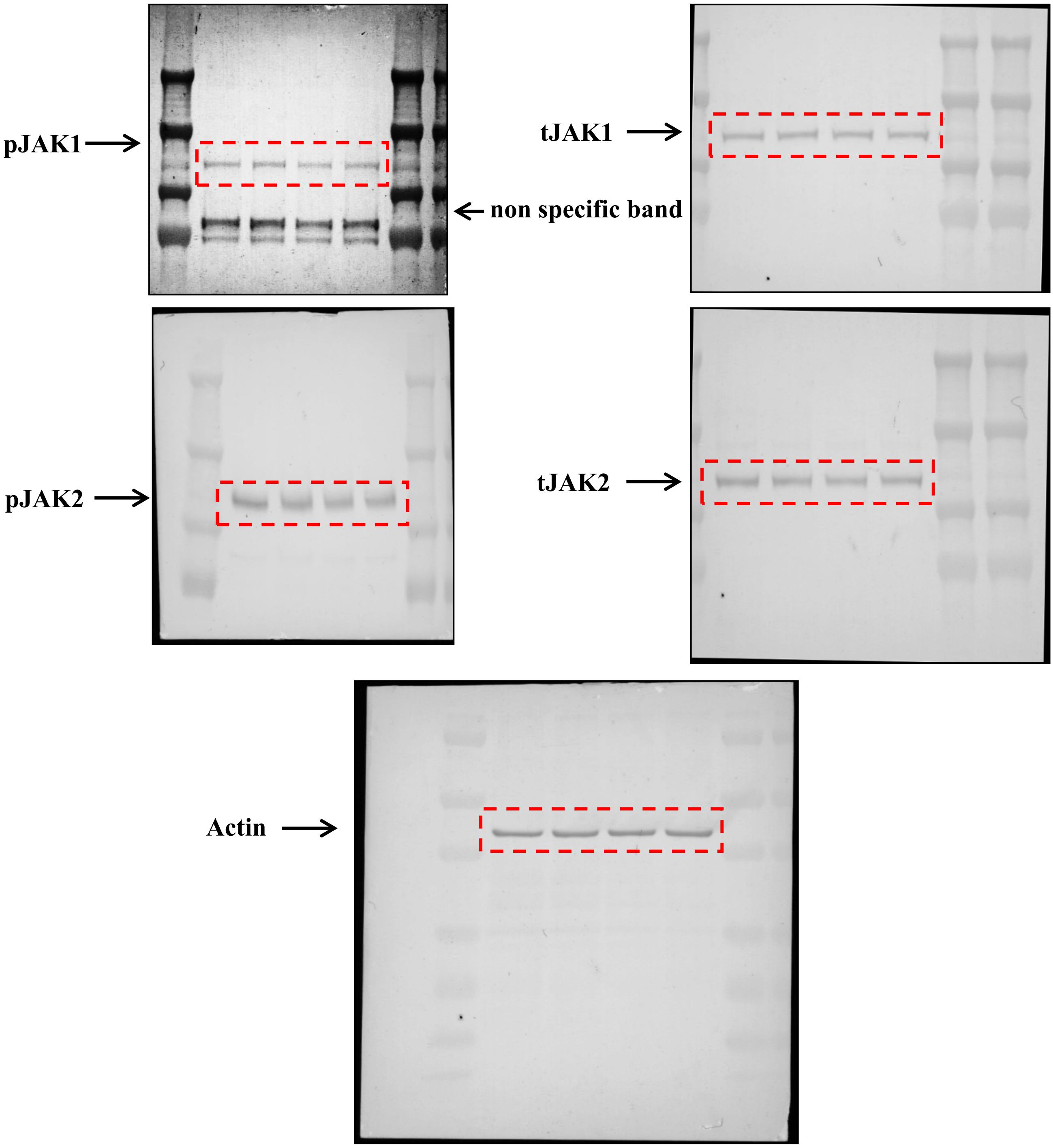

Supplementary Figure S4.
Full-length western blot images of the cropped blots shown in Figure 5.
The hatched red lines delineate the correct sized bands for each protein.

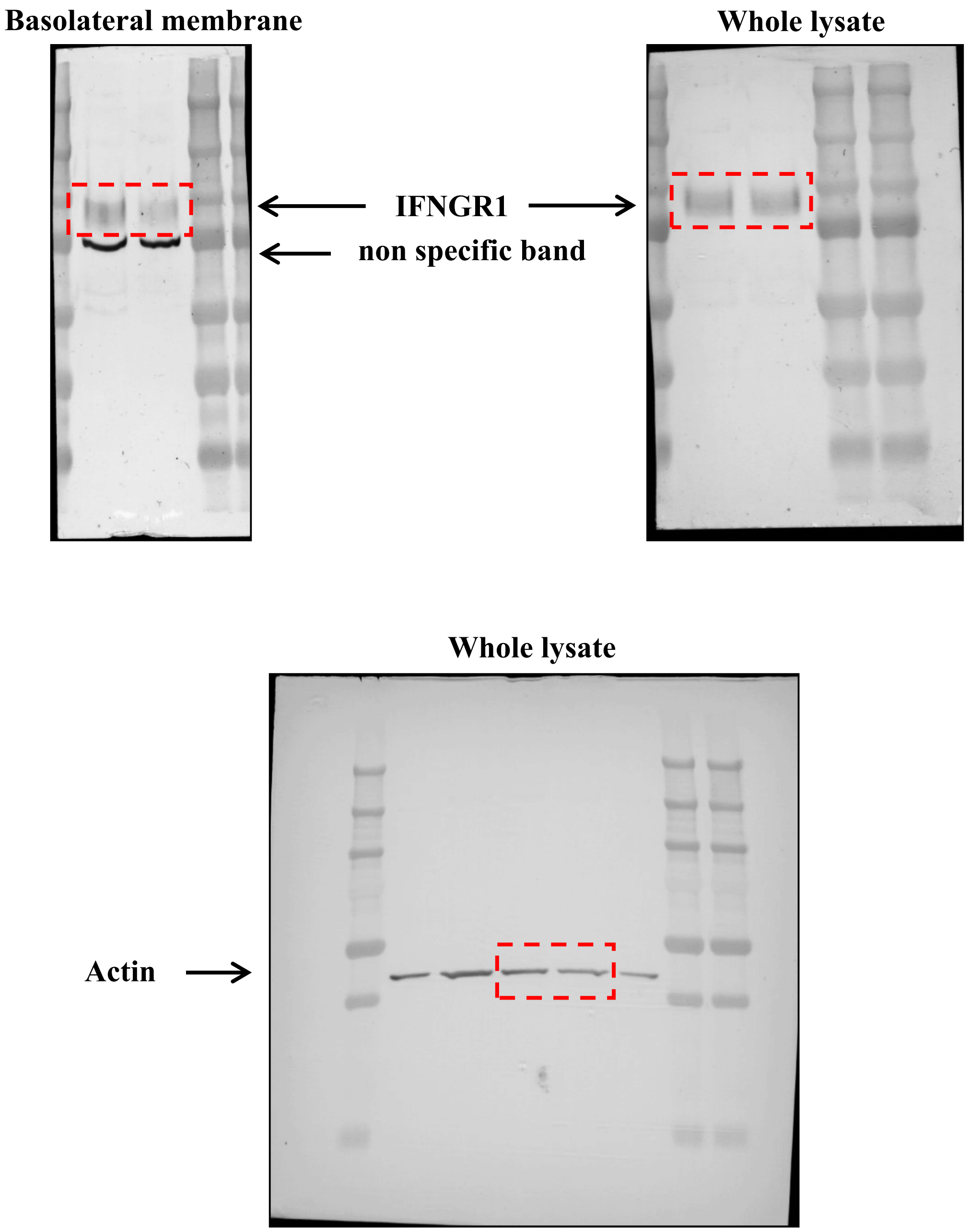

Supplementary Figure S5.

Full-length western blot images of the cropped blots shown in Figure 6.

The hatched red lines delineate the correct sized bands for each protein.

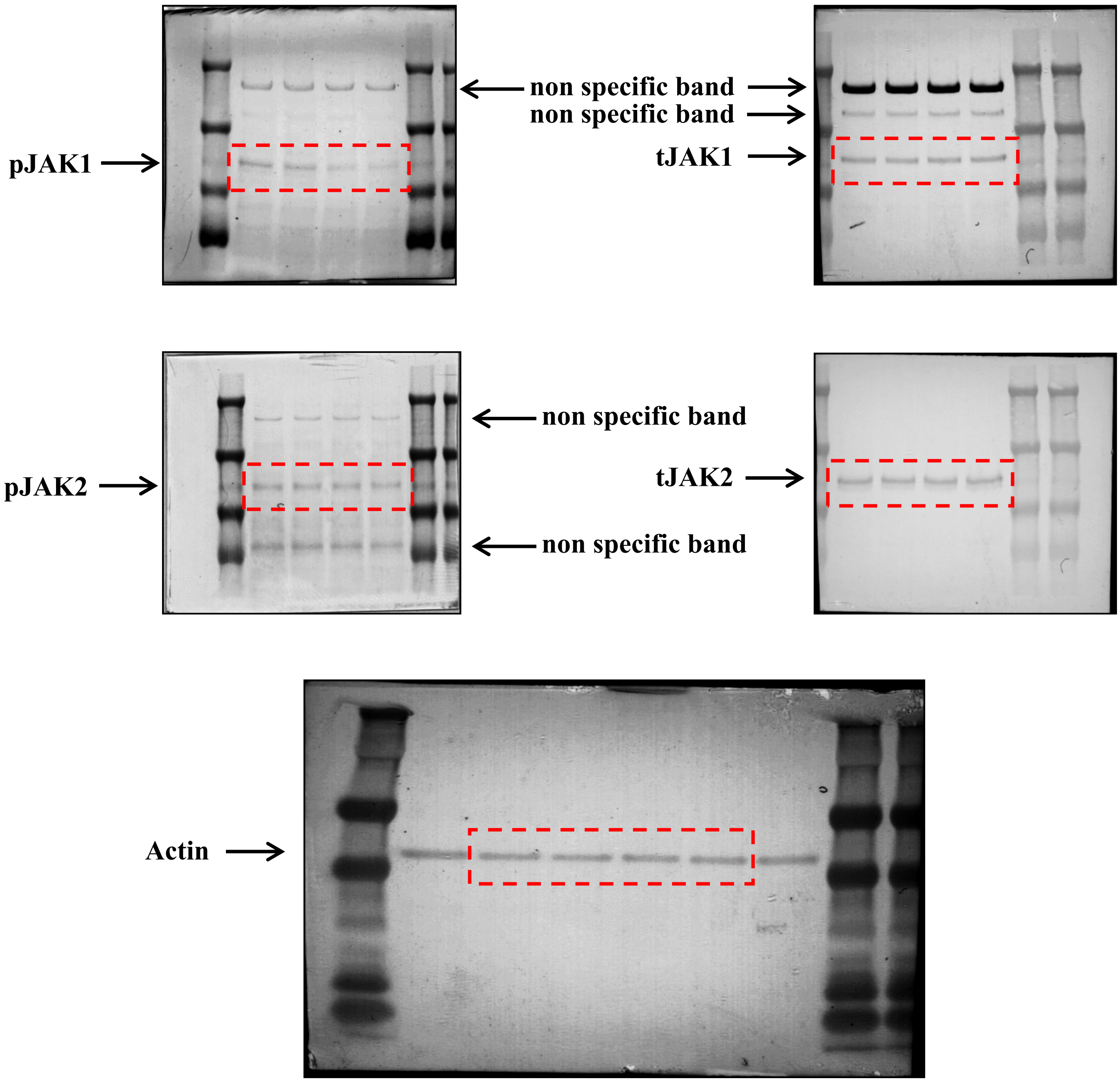

Supplementary Figure S6.
Full-length western blot images of the cropped blots shown in Figure 7.
The hatched red lines delineate the correct sized bands for each protein.

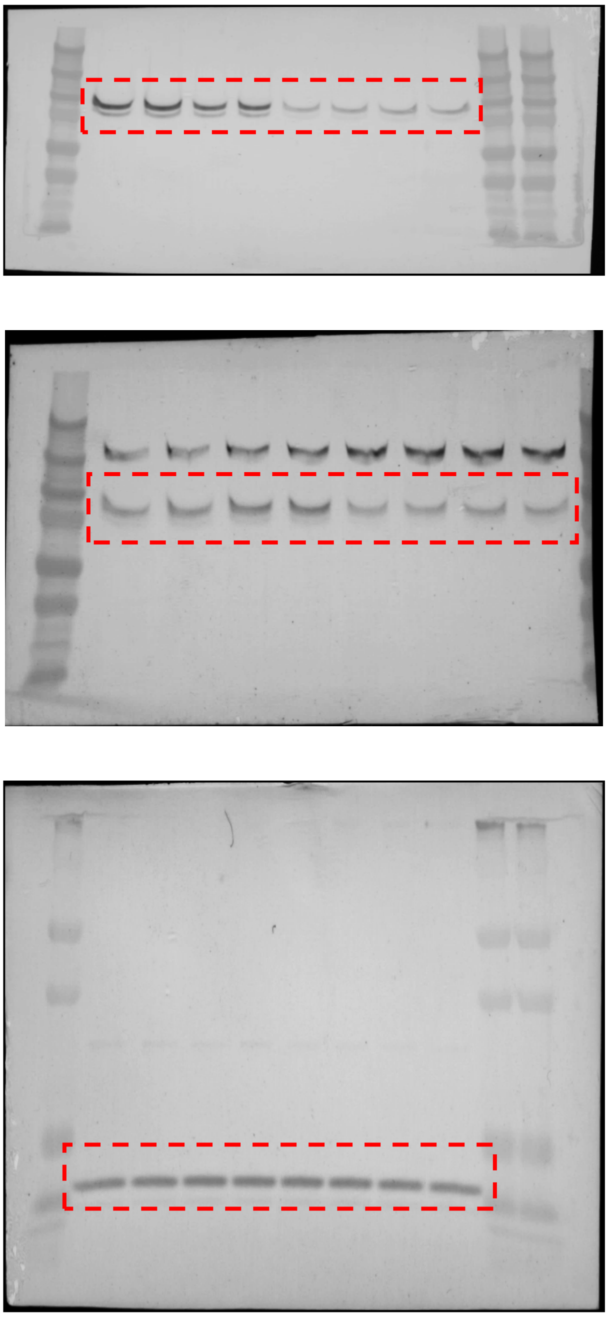

Supplement: Supplementary Information [file srep46580-s1.pdf]
